# Optimized MaxEnt model predicts potential suitable habitats of *Bidens bipinnata* in China under climate change scenario

**DOI:** 10.3389/fpls.2025.1702523

**Published:** 2025-12-16

**Authors:** Yi Liu, Shimao Chen, Hong Zhang, Han Luo, Jiangmiao Hu, Shoujin Liu

**Affiliations:** 1 College of Pharmacy, Anhui University of Chinese Medicine, Hefei, China; 2 Anhui Institute for Food and Drug Control, Hefei, China; 3 Institute of Traditional Chinese Medicine Resources Protection and Development, Anhui Academy of Chinese Medicine, Hefei, China; 4 State Key Laboratory of Phytochemistry and Plant Resources in West China, Kunming Institute of Botany, Chinese Academy of Sciences, Kunming, China

**Keywords:** *Bidens bipinnata*, climate change, environmental variables, MaxEnt model, potential habitat prediction

## Abstract

*Bidens bipinnata*, a traditional Chinese medicinal herb, faces threats from overharvesting and climate change. This study integrated species occurrence data with environmental variables (bioclimatic, soil, and topographic factors). Key variables were selected through correlation analysis and contribution assessment for MaxEnt modeling. The model was optimized by tuning feature combinations and regularization multipliers to achieve high predictive accuracy (AUC > 0.9). The optimized model simulated the potential distribution of suitable habitats under current climate conditions and future scenarios (2030s, 2050s, 2070s, 2090s) for SSP1-2.6 and SSP5-8.5. Changes in suitable area, spatial patterns, and centroid migration were analyzed. The Jackknife test identified July precipitation (prec_07) and February mean temperature (tavg_02) as the dominant factors influencing distribution. Under current conditions, the total suitable area is approximately 1.96 million km^2^, primarily located in central, eastern, and southwestern China. Future projections indicate an overall expansion of suitable habitats, with a trend towards higher latitudes. The distribution centroid, currently in Hubei Province, fluctuates within Hubei under future scenarios, with a more pronounced shift under SSP5-8.5. This study elucidates the ecological drivers and future distribution dynamics of *B. bipinnata*, providing a scientific basis for its resource conservation, cultivation, and sustainable utilization.

## 1 Introduction

*Bidens bipinnata* L., a major botanical source of the genus Bidens in the family Asteraceae, has a long-standing history of medicinal use. It was first documented in the renowned Tang Dynasty medical text *Supplement to Materia Medica (Ben Cao Shi Yi)* by Chen Cangqi. Traditionally, it has been used to clear heat and detoxify, reduce swelling, dissipate stasis, and promote blood circulation. Clinically, it is applied in the treatment of conditions such as the common cold, sore throat, chronic bronchitis, and hepatitis (Zhong et al., 2007; Li et al., 2023). Modern pharmacological studies have revealed that *B. bipinnata* exhibits a range of biological activities, including anti-inflammatory, antioxidant, anticancer, and hepatoprotective effects (Yuan et al., 2008; Wang et al., 2014; Yang et al., 2021). Ecologically, *B. bipinnata* is a common annual herb found in various habitats including roadsides, fields, and thickets. However, due to the growing demand for its medicinal applications and habitat disturbance, the wild resources of *B. bipinnata* have become increasingly scarce (Li et al., 2023), threatening its sustainable availability. Therefore, understanding its ecological requirements and promoting standardized cultivation in climatically suitable regions have become imperative.

Climate change is a major driver altering the geographic distribution patterns of plants worldwide (Parmesan and Yohe, 2003; Liu et al., 2016). It affects species’ adaptability, ecological niches, and distribution ranges. The responses to climate change can differ between woody and herbaceous plants due to variations in dispersal ability, generation time, and physiological plasticity (Corlett and Westcott, 2013). Studying the distribution shifts of medicinal herbs, particularly vulnerable herbaceous species, under climate change is therefore crucial for their conservation and sustainable utilization. Ecological Niche Models (ENMs) are powerful tools that predict species’ potential distributions by quantifying the relationship between known species occurrences and environmental conditions (Peterson et al., 2012; Sofaer et al., 2019). Among various ENMs, the MaxEnt (Maximum Entropy) model has gained widespread application due to its robust performance with presence-only data and ability to handle complex interactions (Phillips et al., 2017; Li et al., 2023). It has been successfully used to project habitat suitability for numerous medicinal plants under climate change, such as *Forsythia suspensa* (Wang et al., 2024), *Angelica dahurica* (Zhang et al., 2024), and *Verbena officinalis* (Chen et al., 2025), informing conservation and cultivation strategies.

Currently, research on *B. bipinnata* primarily focuses on its phytochemistry and pharmacology. Critically, there is a lack of studies investigating the key environmental factors limiting its distribution, modeling its current and future suitable habitats under climate change scenarios, and assessing the congruence between its potential distribution and the specific environmental conditions required for its growth and reproduction. This gap hinders the development of science-based strategies for resource management and cultivation planning.

This study aims to fill this gap by employing an optimized MaxEnt model to predict the potential suitable habitats of *B. bipinnata* across China. Specifically, we seek to address the following questions: (1) What are the dominant environmental factors determining the distribution of *B. bipinnata*? (2) What is the potential geographical distribution of *B. bipinnata* under current climate conditions? (3) How will the suitable habitat area, spatial pattern, and distribution centroid shift under future climate scenarios (SSP1-2.6 and SSP5-8.5) for the periods of the 2030s, 2050s, 2070s, and 2090s? We hypothesize that the distribution of *B. bipinnata* is primarily constrained by specific climatic factors, and that future climate change will lead to significant northward and upward shifts in its suitable habitats. By elucidating the ecological drivers and future distribution dynamics of *B. bipinnata*, this study will provide a scientific basis for the conservation of its wild resources, guide the selection of appropriate regions for its ecological cultivation, and contribute to the sustainable utilization of this important medicinal plant.

## 2 Materials and methods

### 2.1 Collection and processing of distribution data for *B. bipinnata*

**Figure 1:**
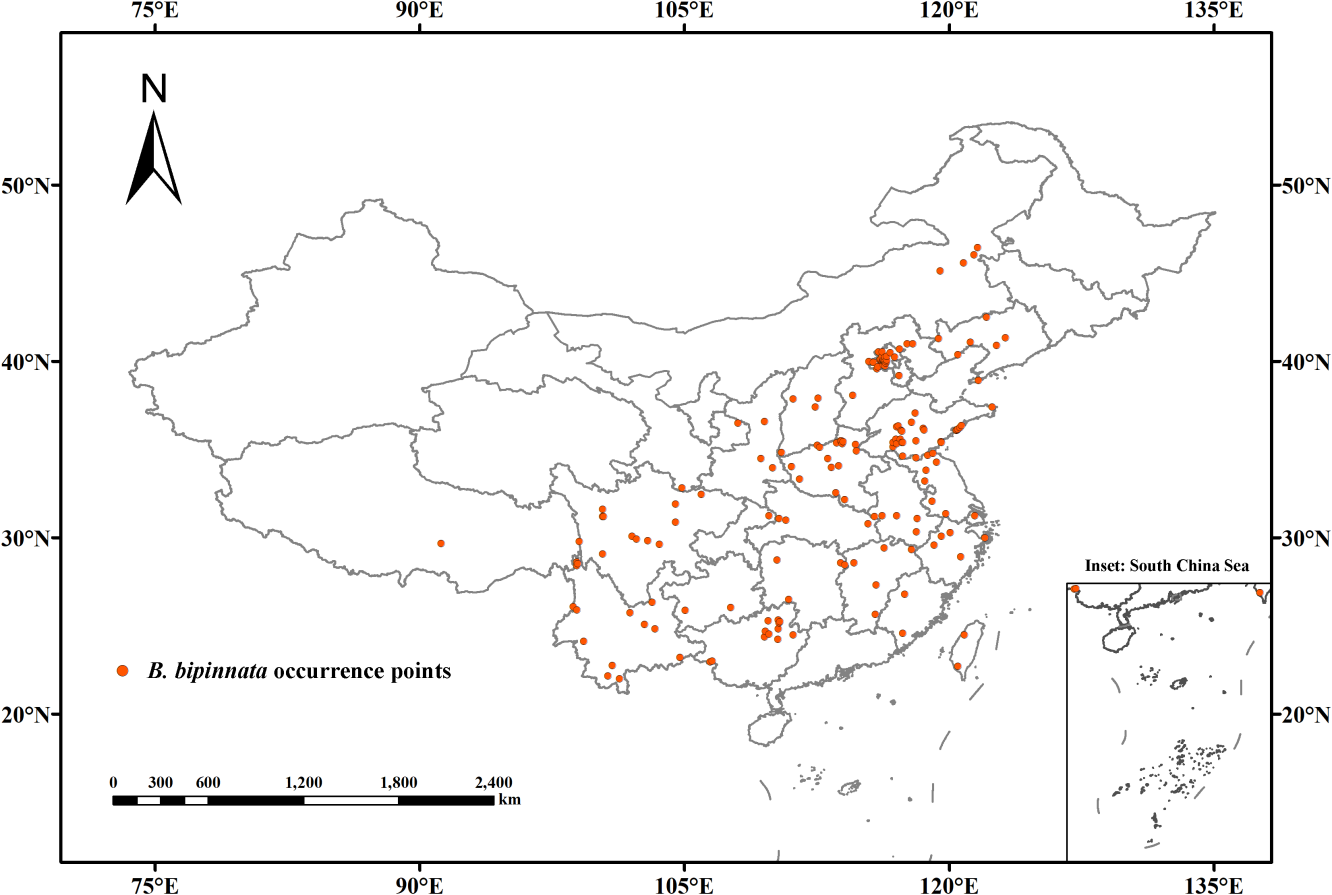
Spatial distribution of (*B*) *bipinnata* occurrence points in China.

Distribution data of *B. bipinnata* were obtained from the Chinese Virtual Herbarium (CVH, 132 records) and the Global Biodiversity Information Facility (GBIF, 54 records). This study exclusively compiled occurrence records within China post-1980 to better align with the temporal scope of the contemporary climate data (1970–2000) from WorldClim, minimizing potential discrepancies caused by significant land-use and climate shifts over longer time spans, yielding an initial dataset of 186 georeferenced points. This study relied on curated records from public databases; accordingly, no original field surveys were conducted by the authors. To mitigate spatial autocorrelation, duplicate or proximate records were filtered using ENMTools software under a 5 km × 5 km grid resolution, a common practice that approximates the typical spatial uncertainty associated with herbarium specimens and reduces overfitting, resulting in 171 spatially independent occurrence points for final analysis (Fu et al., 2024). The spatial distribution of these validated records is presented in **Figure 1**. The inset in the lower-right corner provides a detailed view of the South China Sea region, included to accurately represent the full territorial scope of the study area.

### 2.2 Sources and processing of environmental data

Environmental data used in this study were sourced from multiple repositories. Contemporary and future climate datasets were obtained from WorldClim version 2.1 (Fick and Hijmans, 2017), comprising 19 bioclimatic variables (Bio1–Bio19), monthly precipitation (January–December), and monthly mean temperature (January–December). WorldClim was selected for its widespread use, direct compatibility with CMIP6 future climate projections, and comprehensive set of bioclimatic variables. Three topographic factors (elevation, slope, aspect) were derived from Digital Elevation Model (DEM) data. Soil texture types and associated chemical properties were extracted from the Harmonized World Soil Database (HWSD) v1.2. Future climate projections originated from CMIP6, specifically utilizing the BCC-CSM2-MR model under two contrasting Shared Socioeconomic Pathways (SSP1-2.6 and SSP5-8.5) (Gao et al., 2023). Four future periods were evaluated: 2030s (2021–2040 mean), 2050s (2041–2060 mean), 2070s (2061–2080 mean), and 2090s (2081–2100 mean). All 66 environmental variables were standardized to a 2.5-minute spatial resolution (~5 km^2^ at the equator) and projected to the WGS84 geographic coordinate system to ensure spatial alignment. No additional spatial interpolation was performed beyond the native processing of the source datasets. Detailed descriptions of all 66 initial variables are provided in **Supplementary Table S1**.

**Figure 2:**
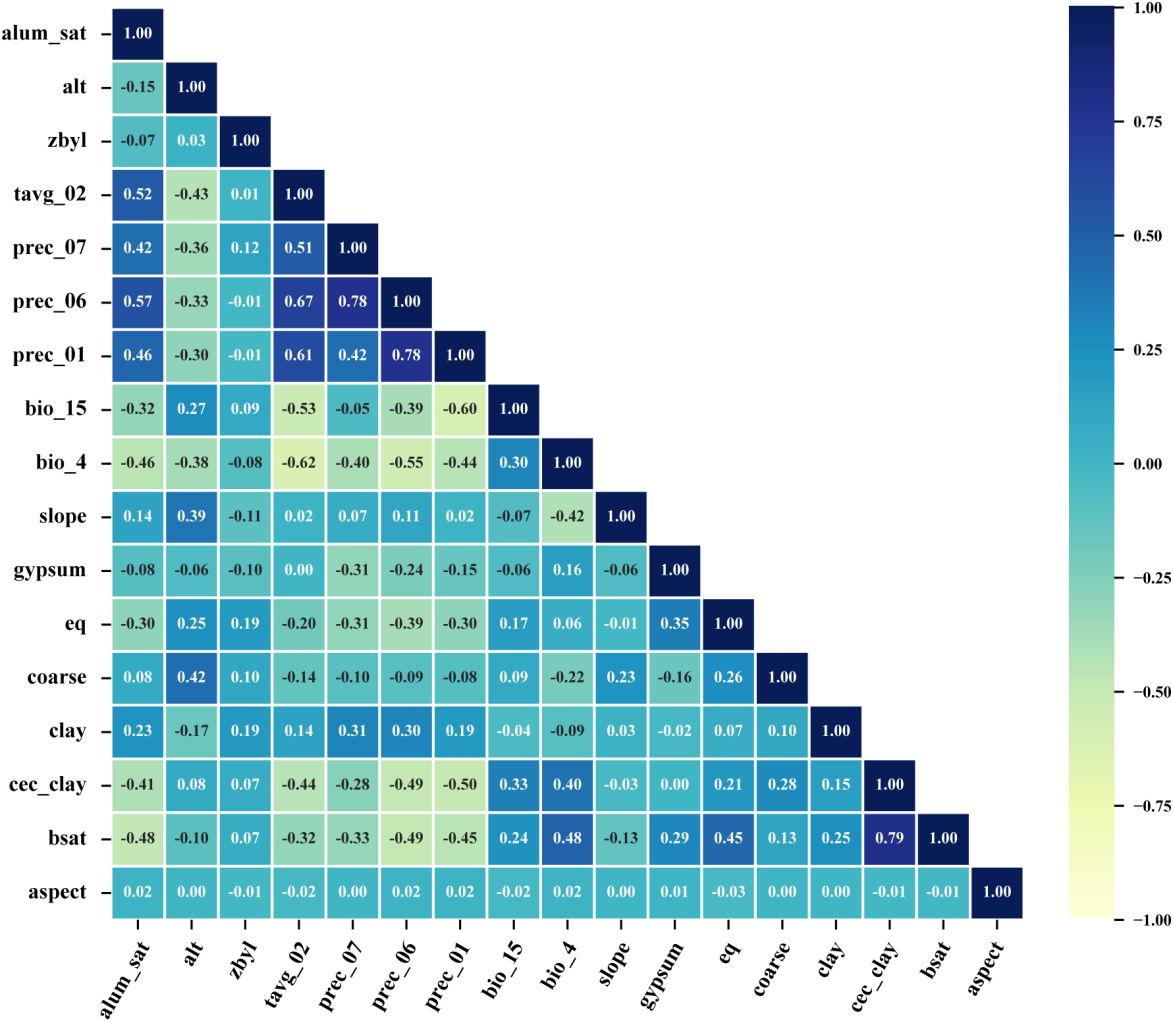
The correlation among the 17 environmental variables selected for MaxEnt modeling.

Given potential multicollinearity among environmental predictors, all 66 variables were initially incorporated into a preliminary MaxEnt run alongside 171 occurrence points to quantify variable contribution rates. Concurrently, pairwise Pearson correlation coefficients (|r|) were calculated using ENMTools. To select a robust and parsimonious set of predictors, we adopted a two-step filtering strategy: First, from any pair of variables with |r| ≥ 0.8, we retained the one with the higher contribution rate in the preliminary model to mitigate multicollinearity. Second, we retained variables with a permutation importance greater than zero, ensuring they provided non-random information to the model. This dual-filtering approach yielded 17 key predictors for subsequent modeling, optimization, and evaluation (**Table 1**). The categorical variable “zbyl” (vegetation classification index) was included as it reflects broad ecosystem types and associated microclimates and soil conditions and was converted to a numerical raster for processing. A correlation matrix for the final 17 variables is provided in **Figure 2**, confirming the absence of strong multicollinearity (|r| < 0.8) among them.

**Table 1:** Detailed information on the 17 environmental variables.

Variable code	Environmental factor	Unit
prec_07	Precipitation in July	mm
tavg_02	February mean temperature	°C
alum_sat	Aluminium saturation	%
eq	Calcium Carbonate	%
zbyl	Vegetation Classification	
slope	Slope	°
bio_4	Temperature Seasonality	°C×100
bio_15	Precipitation Seasonality	%
bsat	Base Saturation	%
aspect	Aspect	rad
alt	Altitude	m
cec_clay	CEC clay	cmolc/kg
prec_06	Precipitation in June	mm
clay	Clay	%
gypsum	Gypsum content	%
coarse	Coarse fragments	%
prec_01	Precipitation in January	mm

### 2.3 Establishment, optimization, and evaluation of the MaxEnt model

Processed occurrence data of *B. bipinnata* were saved in CSV format and imported into MaxEnt alongside the filtered environmental variables. Model parameters were configured as follows: 25% of occurrence data were allocated to the test set, 75% to the training set; the Bootstrap method was applied with a default maximum of 10,000 background points; random seed selection was enabled to ensure reproducibility; 10 replicates were executed; and the output format was set to logistic.

Further, given the sensitivity of MaxEnt predictions to the regularization multiplier (RM), feature combination (FC), and maximum background points, comprehensive parameter optimization was conducted using the Kuenm package in R 4.4.1. Five feature classes were evaluated: linear (L), quadratic (Q), hinge (H), product (P), and threshold (T). These were combined into 31 distinct FC settings (default: LQPH). RM values spanned 0.1 to 4.0 at 0.5 intervals (8 levels). All 248 parameter combinations (31 FC × 8 RM) were tested using 75% training data. Model performance was assessed via: (1) statistical significance (partial ROC, p < 0.05), (2) omission rate (E = 5%, signifying that models predicted at least 95% of test points correctly), and (3) model complexity (lowest AICc). From all candidate models that met the first two criteria, the model with the lowest AICc value was selected as optimal (Muscarella et al., 2014). The optimal parameter set was subsequently applied to establish the final model. We note that MaxEnt, like most correlative ENMs, assumes equilibrium between species distributions and current climate, an assumption that may be violated under rapid climate change (Street, 2020); this is an inherent limitation of our projection approach.

The area under the receiver operating characteristic curve (AUC) quantifies model accuracy, with values ranging from 0 to 1. Higher AUC values indicate superior predictive performance, unaffected by prevalence in sample data. Interpretation follows established thresholds: 0.5–0.6 (model failure), 0.6–0.7 (poor), 0.7–0.8 (moderate), 0.8–0.9 (good), and 0.9–1 (excellent). In this study, AUC values evaluated model efficacy, where higher scores reflected stronger correlations between the predicted distribution of *B. bipinnata* and environmental drivers, confirming robust predictive capability (Ma et al., 2021).

### 2.4 Data processing for the execution of the MaxEnt model

To quantify changes in suitable habitat area for *B. bipinnata* under current and future climate scenarios, habitat suitability layers were classified and visualized using ArcGIS 10.8.1. The maximum test sensitivity plus specificity (MTSPS) threshold—derived directly from MaxEnt outputs by balancing model sensitivity and specificity—was employed to delineate suitability zones. Habitats were categorized as: non-suitable (0–MTSPS), low-suitability (MTSPS–0.5), medium-suitability (0.5–0.7), and high-suitability (0.7–1). Areal extents of each zone were computed in ArcGIS.

Spatiotemporal shifts were analyzed through binary classification: areas below MTSPS were deemed unsuitable, while those ≥ MTSPS were designated suitable. Future habitat changes relative to the current baseline were defined as: (1) range expansion (newly suitable areas), (2) range contraction (currently suitable areas becoming unsuitable), and (3) range retention (persistently suitable areas). The geometric centroid of suitable habitats—representing the spatial nucleus of the species’ distribution—was calculated for each period using ArcGIS’s Zonal Geometry tool (Lenoir et al., 2008). Assuming unlimited dispersal capacity and neglecting interspecific interactions, centroid migration trajectories (direction and distance between consecutive periods) were generated as vector files to characterize shifts under varying climate scenarios (Li et al., 2019).

## 3 Results

### 3.1 Model optimization and accuracy evaluation

**Figure 3:**
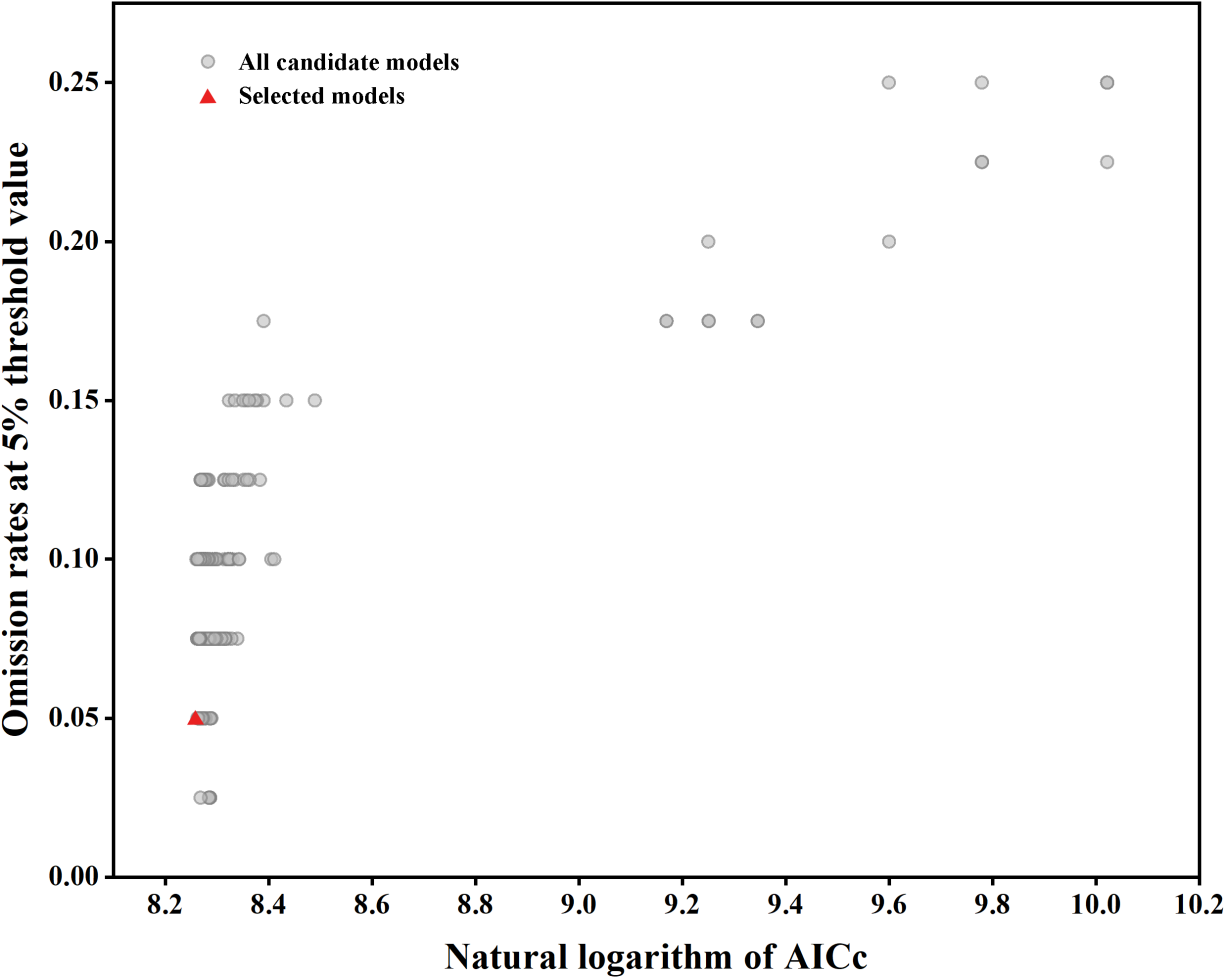
MaxEnt model parameter optimization results.

The optimized MaxEnt model (FC = PH, RM = 4) demonstrated superior performance with a mean AUC of 0.924 ± 0.012 (mean ± SD from 10 replicates) and a low omission rate of 5.2%. In contrast, the default model (FC = LQPH, RM = 1) had a mean AUC of 0.901 ± 0.015 and an omission rate of 7.8%. The AICc value for the optimized model was 3873.46, significantly lower than that of the default model (4197.06), indicating better fit with reduced complexity (**Figure 3**, **Table 2**). These results confirm the selection of the optimized parameters for subsequent analysis.

**Table 2:** Performance evaluation of MaxEnt model under initial and optimized parameters.

Model evaluation	Feature combination	Regularization multiplier	Omission rate (%)	Mean AUC ratio	AICc
Default	LQPH	1	7.8	1.733	4197.06
Optimized	PH	4	5.2	1.605	3873.46

### 3.2 The influence of key environmental variables on the distribution of *B. bipinnata*

**Figure 4:**
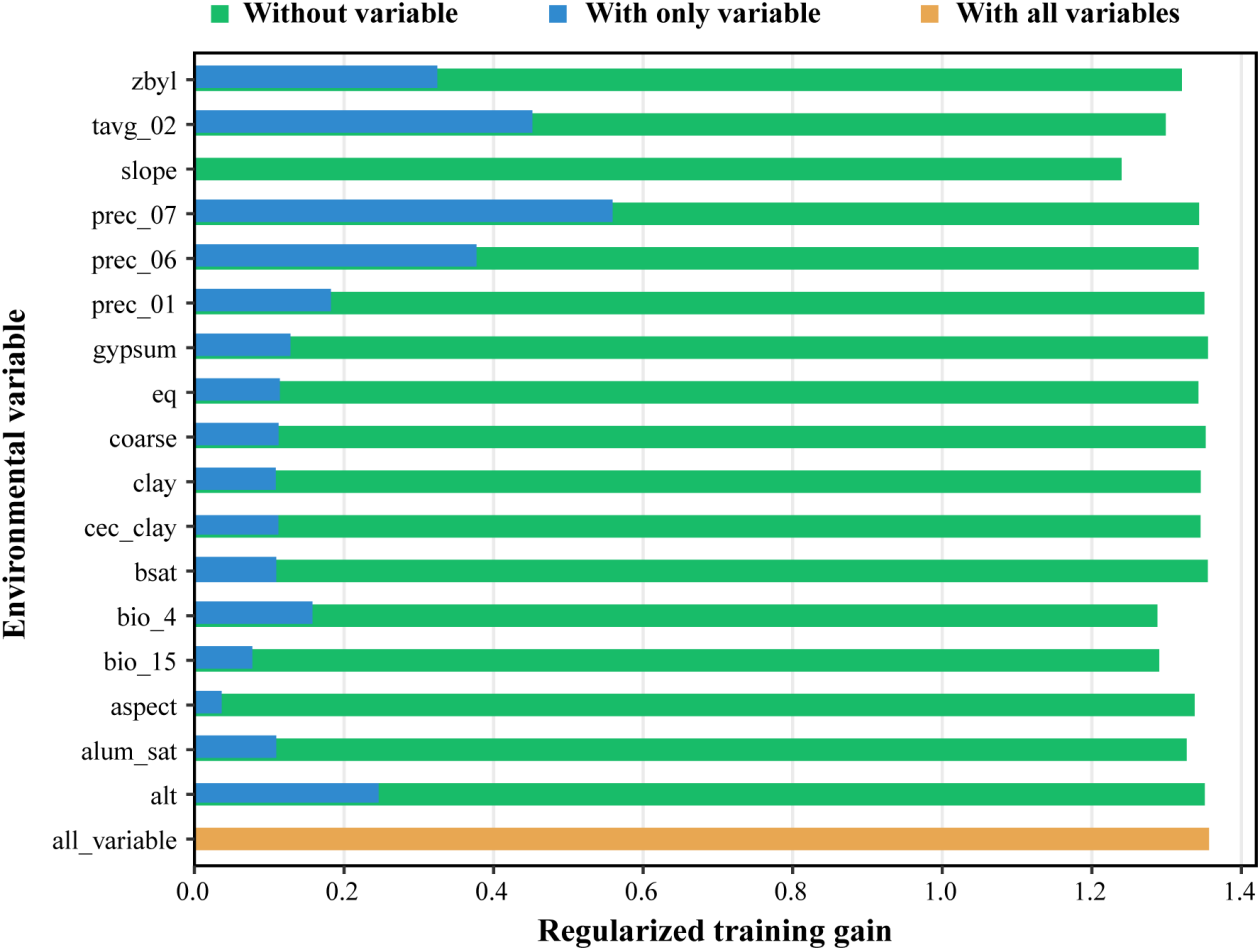
Results of jackknife test for the importance of the variables for MaxEnt.

**Figure 5:**
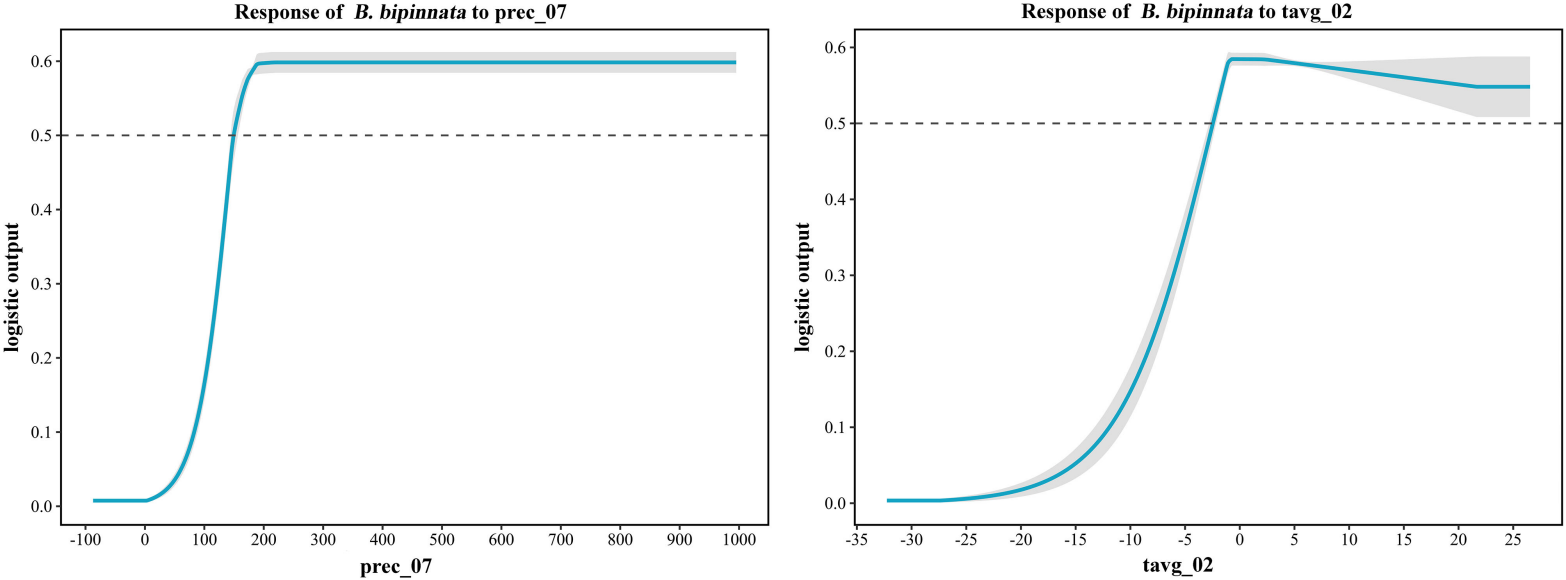
Response curves of key environmental variables (prec_07, tavg_02) influencing the suitable distribution of (*B*) *bipinnata.*.

Analysis of 17 key environmental variables revealed that precipitation in July (prec_07) and mean February temperature (tavg_02) were the primary determinants, with contribution rates of 36.4% and 20.9%, respectively, and a cumulative contribution of 57.3% (**Table 3**). Jackknife validation further confirmed the dominance of prec_07 and tavg_02, as both yielded the highest regularized training gains when modeled independently (**Figure 4**). These factors are biologically critical: July precipitation supports flowering and fruiting, while February temperature influences overwintering and early growth. Other variables, such as aluminum saturation (alum_sat, 8%), calcium carbonate equivalent (eq, 7.7%), and vegetation classification (zbyl, 7.6%), also played notable roles. Soil variables like alum_sat and eq may affect nutrient availability and root development. Response curves indicated optimal ranges of 149.85–962 mm for July precipitation and -2.45–22.47 °C for February mean temperature (**Figure 5**). Extreme values beyond these ranges should be interpreted with caution due to potential model extrapolation.

**Table 3:** The contribution rate of environmental variables.

Variable code	Environmental factor	Unit	Percent contribution/%	Permutation importance/%
prec_07	Precipitation in July	mm	36.4	7.2
tavg_02	February mean temperature	°C	20.9	37.3
alum_sat	Aluminium saturation	%	8	5
eq	Calcium Carbonate	%	7.7	5.5
zbyl	Vegetation Classification		7.6	2.7
slope	Slope	°	4	7.1
bio_4	Temperature Seasonality	°C×100	3.9	12.2
bio_15	Precipitation Seasonality	%	3.1	8.1
bsat	Base Saturation	%	2	0.2
aspect	Aspect	rad	2	1.5
alt	Altitude	m	1.4	3.1
cec_clay	CEC clay	cmolc/kg	1.1	0.8
prec_06	Precipitation in June	mm	0.8	5.4
clay	Clay	%	0.5	0.5
gypsum	Gypsum content	%	0.2	0.2
coarse	Coarse fragments	%	0.2	0.5
prec_01	Precipitation in January	mm	0.2	2.8

### 3.3 The suitable distribution of *B. bipinnata* under current climatic conditions

**Figure 6:**
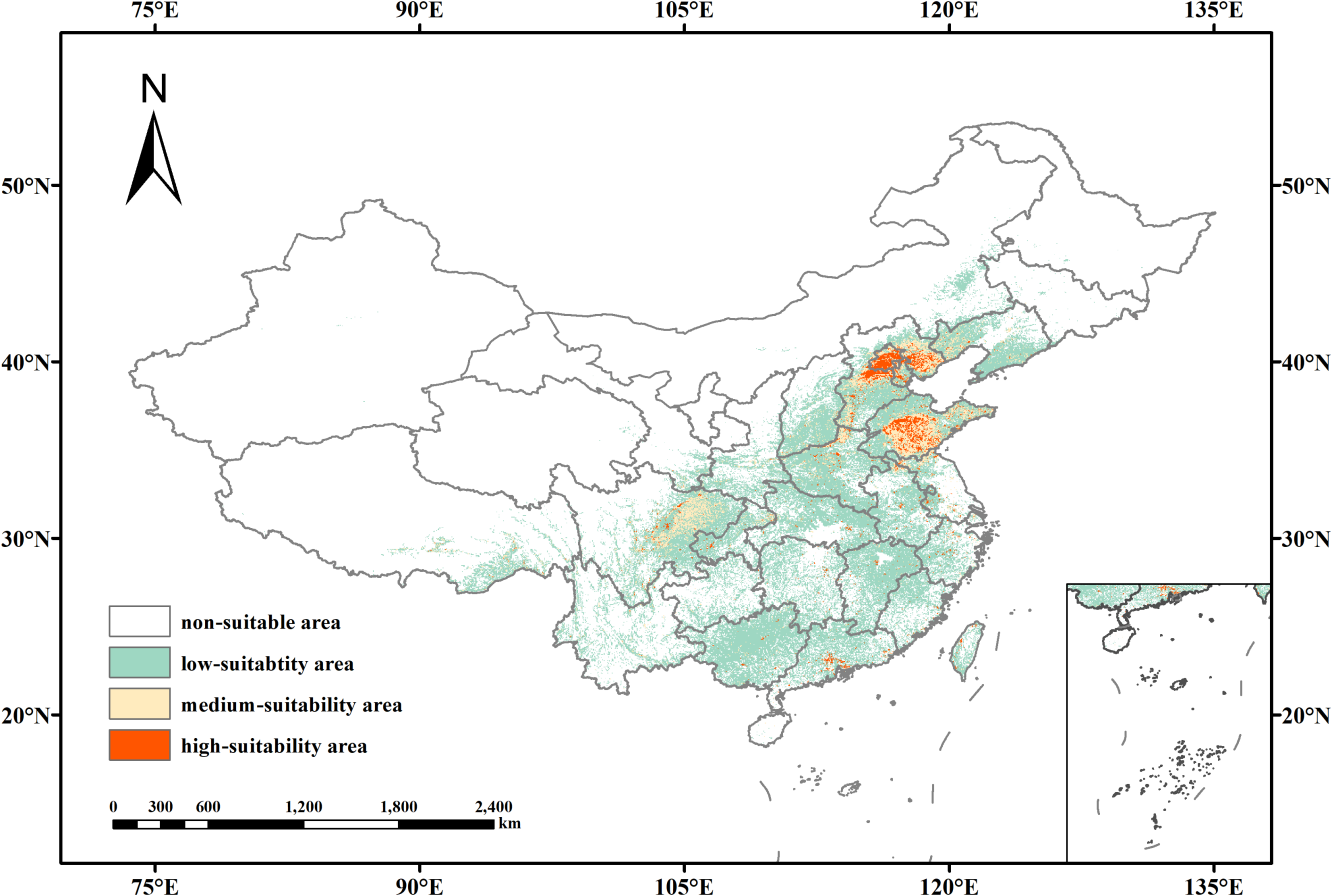
Potential suitable distribution of (*B*) *bipinnata* under current climatic conditions.

According to the MaxEnt model outputs, the potential suitable habitat for *B. bipinnata* in China under current climatic conditions was classified into four categories based on habitat suitability scores: unsuitable (0–0.2163), low suitability (0.2163–0.5), moderate suitability (0.5–0.7), and high suitability (0.7–1.0) (**Figure 6**). The total suitable habitat area was estimated at approximately 1.96 × 10^6^ km^2^, accounting for 20.33% of China’s terrestrial surface. These suitable areas are primarily distributed across Central China, Eastern China, Southwestern China, and parts of the Southern region. The highly suitable habitats are predominantly concentrated around the central region of Henan Province, western Shandong, southern Hebei, and northern Anhui, with additional minor zones in north-central Fujian and central Taiwan. The total area of high suitability habitat amounts to 8.36 × 10^4^ km^2^ representing 0.87% of China’s land area. Moderately suitable areas are more broadly distributed, primarily across northeastern Yunnan, central-eastern Guizhou, southern Sichuan, southwestern Hunan, and northern Guangxi, with additional presence in western Hubei, southern Shaanxi, and central Anhui. These zones encompass approximately 2.36 × 10^5^ km^2^, accounting for 2.45% of the national land area. Low suitability zones exhibit the broadest geographic coverage, encompassing large portions of southwestern, southern, central, and eastern China, including provinces such as Yunnan, Guizhou, Sichuan, Guangxi, Hunan, Jiangxi, Fujian, Guangdong, Chongqing, and Hubei. These areas collectively span 1.64 × 10^6^ km^2^, constituting 17.01% of the country’s total landmass.

### 3.4 The suitable distribution of *B. bipinnata* under future climate conditions

**Figure 7:**
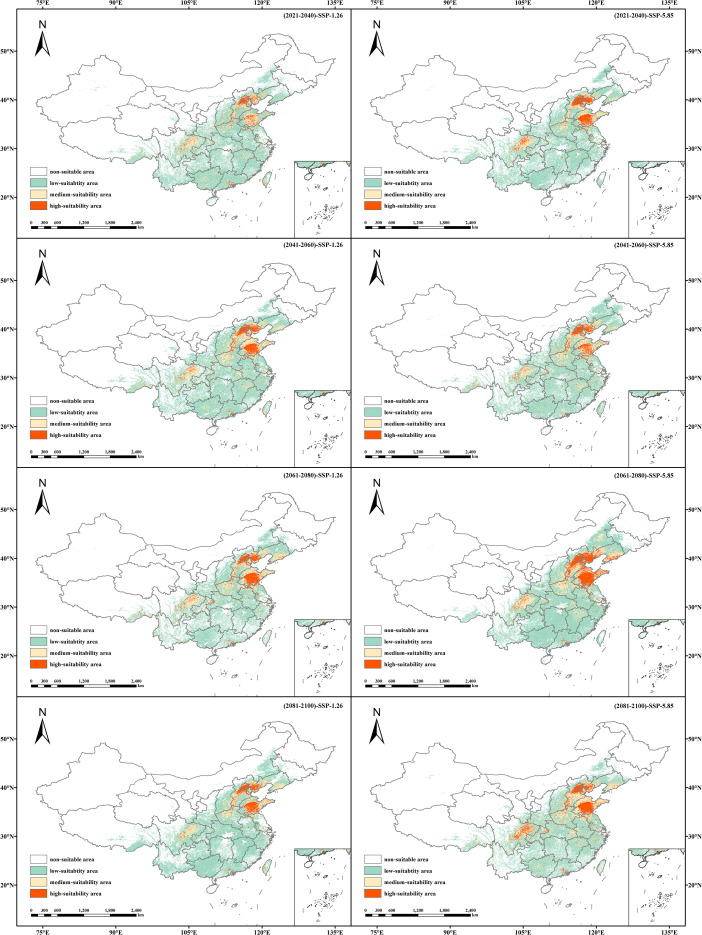
Suitable habitat distribution of (*B*) *bipinnata* under different future climate scenarios (SSP1-2.6 and SSP5-8.5).

Based on MaxEnt model projections under two emission scenarios across four future periods (2020–2040, 2041–2060, 2061–2080, and 2081–2100), the potential distribution of suitable habitats for *B. bipinnata* in China was estimated, along with the corresponding habitat suitability classifications using the MTSPS threshold (**Table 4**, **Figure 7**).

**Table 4:** Area of suitable habitats for *B. bipinnata* under current and future climate scenarios by suitability levels.

Decade scenarios	Predicted area (× 10^4^ km^2^)				
	Low habitat suitability	Medium habitat suitability	High habitat suitability	Unsuitable habitat	Total suitable area
Current	163.69	23.59	8.36	766.71	195.64
2030s-SSP1-2.6	203.72	41.35	11.80	705.48	256.87
2050s-SSP1-2.6	202.62	55.11	16.95	687.68	274.68
2070s-SSP1-2.6	162.67	48.83	19.26	731.59	230.76
2090s-SSP1-2.6	207.73	35.80	15.67	703.16	259.20
2030s-SSP5-8.5	189.27	37.45	16.13	719.50	242.85
2050s-SSP5-8.5	195.10	51.24	15.94	700.08	262.28
2070s-SSP5-8.5	211.48	52.66	27.91	670.30	292.05
2090s-SSP5-8.5	174.38	63.47	22.76	701.74	260.62

Under the SSP1-2.6 scenario, the total area of suitable habitat for *B. bipinnata* is projected to increase compared to current climatic conditions. The largest extent is observed during the 2050s, reaching 2.75 × 10^6^ km^2^—an increase of 6.4 × 10^5^ km^2^ relative to the present. Notably, the area classified as highly suitable increases by 8.59 × 10^4^ km^2^, representing approximately a twofold expansion. The moderately and lowly suitable areas expand by 3.15 × 10^5^ km^2^ and 3.89 × 10^5^ km^2^, respectively.

Under the SSP5-8.5 scenario, a similar trend of increasing habitat suitability is observed, with the total suitable area peaking in the 2070s at 2.92 × 10^6^ km^2^—6.4 × 10^5^ km^2^ more than under current conditions. The high suitability zone exhibits the most substantial relative increase, expanding by 1.94 × 10^5^ km^2^, which is approximately three times the current extent. Moderate and low suitability areas also show marked expansion, increasing by 2.91 × 10^5^ km^2^ and 4.78 × 10^5^ km^2^, respectively, compared to present-day distributions.

Under SSP1-2.6, the maximum expansion occurred in the 2050s (2.75 × 10^6^ km^2^), with high-suitability areas doubling. Under SSP5-8.5, the peak was in the 2070s (2.92 × 10^6^ km^2^), with high-suitability areas tripling. These shifts indicate a general northward and upward migration in response to climate warming.

### 3.5 Spatial pattern changes in the future potential distribution of *B. bipinnata*

**Figure 8:**
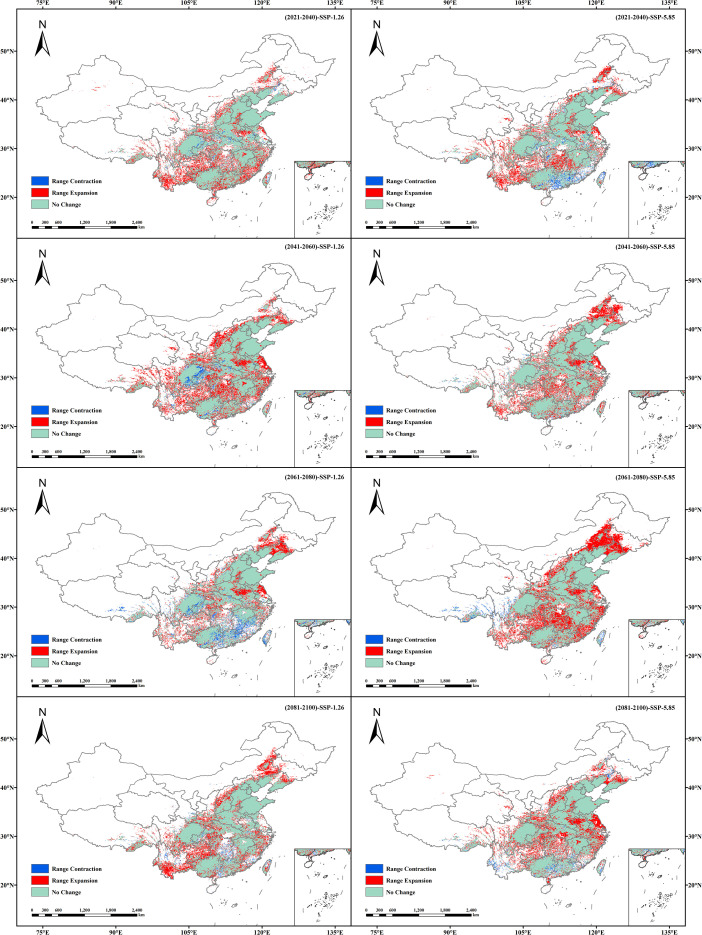
Spatial pattern changes in the potential suitable habitats of (*B*) *bipinnata* under different future climate scenarios.

Under contrasting climate change scenarios, *B. bipinnata* exhibits distinct spatiotemporal shifts in its potential suitable habitats (**Figure 8**). Under the SSP1-2.6 scenario, the overall spatial distribution of suitable habitats remains relatively stable, with moderate spatial turnover between expansion and contraction zones. Over the period from the 2030s to the 2090s, the expansion area fluctuates slightly from 7.13 × 10^5^ km^2^ to 7.33 × 10^5^ km^2^. The areas of expansion are mainly concentrated at the peripheries of the current suitable zones, with gradual advancement toward northern provinces and certain high-altitude regions such as the western Sichuan Plateau and the Qinling–Daba Mountains. Meanwhile, stable habitat zones dominate the spatial pattern, ranging between 1.78 × 10^6^ and 1.90 × 10^6^ km^2^. Habitat loss remains limited, with area loss increasing slightly from 5.80 × 10^4^ km^2^ to a peak of 1.78 × 10^5^ km^2^ before declining to 6.09 × 10^4^ km^2^. These reductions primarily occur in ecologically marginal areas such as eastern Sichuan Basin, central Hubei, and southeastern Tibet.

In contrast, under the SSP5-8.5 scenario, the expansion trend of potential suitable habitat for *B. bipinnata* intensifies significantly, with marked shifts toward northern high-latitude regions and the northwestern plateau. The expansion area increases progressively from 6.26 × 10^5^ km^2^ in the 2030s to 9.43 × 10^5^ km^2^ in the 2050s, reaching a maximum of 9.92 × 10^5^ km^2^ in the 2070s. These expansions predominantly occur in the northern North China Plain, western Northeast China, northern edge of the Loess Plateau, and eastern margins of the Qinghai–Tibet Plateau. Stable suitable zones remain in the range of 1.75 × 10^6^ to 1.86 × 10^6^ km^2^maintaining the core distribution despite minor fluctuations. The area of habitat contraction shows a trend of initial decline followed by a rebound, decreasing from 1.18 × 10^5^ km² in the 2030s to 6.75 × 10^4^ km^2^ in the 2070s, then increasing to 1.23 × 10^5^ km^2^ in the 2090s. These reductions are mainly localized in transitional climate zones such as central Henan, the periphery of the Sichuan Basin, and southern Shaanxi.

### 3.6 Centroid migration of suitable habitats in future periods

**Figure 9:**
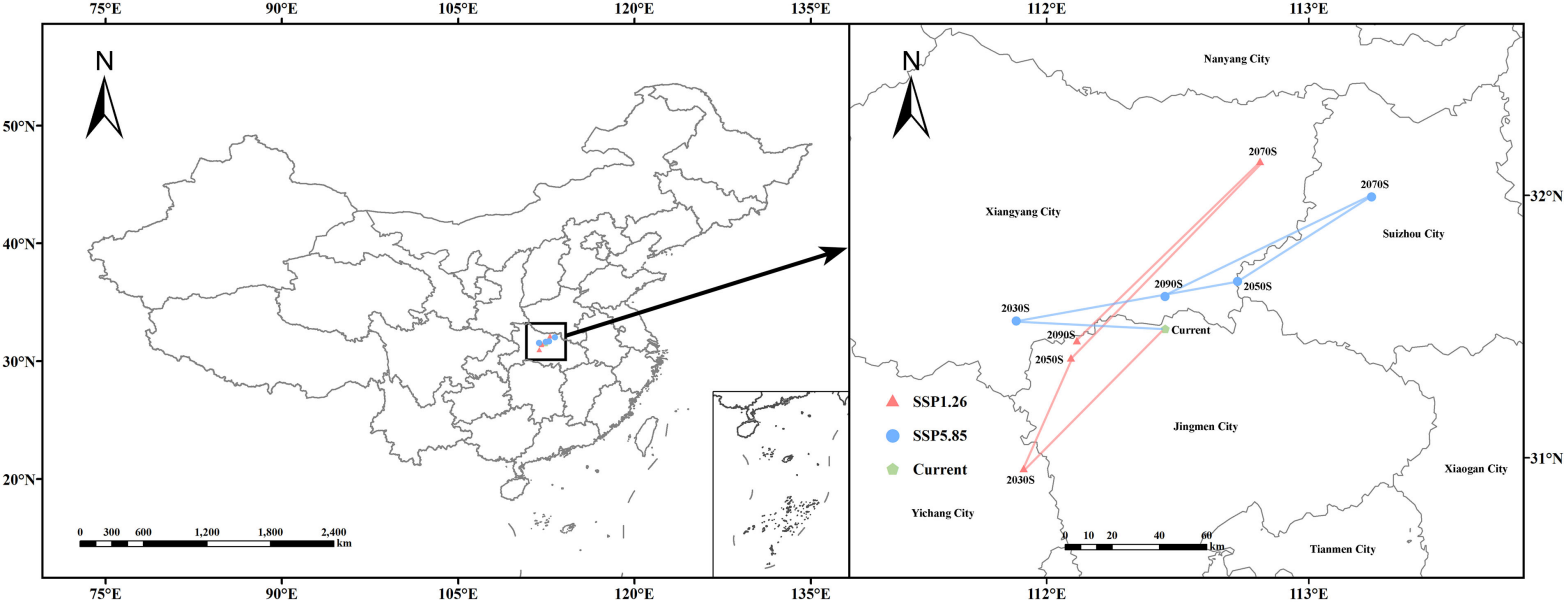
Migration trajectory of the geometric centroid of suitable areas for (*B*) *bipinnata* under future climate scenarios.

Using the MTSPS value (MTSPS = 0.2163) as the threshold, the suitable and unsuitable areas for *B. bipinnata* were classified. Based on this classification, the temporal migration of habitat suitability centroids under SSP1-2.6 and SSP5-8.5 emission scenarios were analyzed using ArcGIS, and the trajectories of centroid shifts were visualized (**Table 5**, **Figure 9**). Under current climatic conditions, the centroid of the suitable habitat is located in Zhongxiang City, Jingmen, Hubei Province (112.45°E, 31.49°N). Under the SSP1-2.6 scenario, during 2021–2040, the centroid shifted 78.5 km southwest to Dangyang City, Yichang, Hubei Province (111.91°E, 30.96°N). In 2041–2060, it moved 49.99 km northeast to Dongbao District, Jingmen (112.09°E, 31.38°N); in 2061–2080, it further migrated 107.71 km northeast to Zaoyang City, Xiangyang, Hubei (112.82°E, 32.13°N); and in 2081–2100, it shifted 100.76 km southwest back to Dongbao District, Jingmen (112.12°E, 31.45°N).

**Table 5:** Centroid migration of suitable habitats for *B. bipinnata* under future climate scenarios.

Scenario	Period	Longitude (°E)	Latitude (°N)	Distance (km)
Current	–	112.45	31.49	–
SSP1-2.6	2021–2040	111.91	30.96	78.50
SSP1-2.6	2041–2060	112.09	31.38	49.99
SSP1-2.6	2061-2080	112.82	32.13	107.71
SSP1-2.6	2081-2100	112.12	31.45	100.76
SSP5-8.5	2021–2040	111.88	31.52	54.33
SSP5-8.5	2041–2060	112.73	31.67	82.1
SSP5-8.5	2061-2080	113.24	31.99	60.29
SSP5-8.5	2081-2100	112.45	31.61	85.84

Under the SSP5-8.5 scenario, the centroid moved 54.33 km northwest to Nanzhang County, Xiangyang, Hubei (111.88°E, 31.52°N) during 2021–2040. It then shifted 82.1 km northeast to Suixian County, Suizhou, Hubei (112.73°E, 31.67°N) during 2041–2060. From 2061–2080, the centroid continued its northeastward migration within Suixian County, moving 60.29 km to 113.24°E, 31.99°N. Finally, in 2081–2100, the centroid moved 85.84 km southwest to Yicheng City, Xiangyang, Hubei Province (112.45°E, 31.61°N). The assumption of unlimited dispersal may overestimate expansion; actual migration could be slower due to biological and environmental constraints.

## 4 Discussion

### 4.1 Methodological advances and model reliability

This study demonstrates the value of systematically optimizing MaxEnt parameters to improve the reliability of species distribution projections under climate change. By refining feature classes and regularization multipliers, we achieved a model that balances complexity and generalizability, as evidenced by the lower AICc and omission rate compared to the default configuration. Our approach aligns with recent efforts to enhance the robustness of ecological niche models (Seo et al., 2021), particularly for medicinal plants with specific habitat requirements. The use of the MTSPS threshold further strengthens habitat classification by minimizing subjective bias (Zhang et al., 2018). These methodological refinements are critical for translating model outputs into actionable conservation and cultivation strategies.

### 4.2 Key environmental drivers and their ecophysiological basis

Our analysis identified July precipitation (prec_07) and February mean temperature (tavg_02) as the dominant factors shaping the distribution of *B. bipinnata*, together accounting for 57.3% of the model’s explanatory power. This finding is consistent with studies on other herbaceous medicinal plants, such as *Cirsium lineare* (Fang et al., 2024) and *Verbena officinalis* (Chen et al., 2025), where seasonal climate variables strongly influenced habitat suitability. Ecophysiologically, July represents a critical period for flowering and fruit development in *B. bipinnata*, and adequate precipitation during this phase likely supports successful reproduction. Conversely, February temperature influences overwintering survival and the timing of spring germination; milder conditions may facilitate earlier growth resumption, while extreme cold could limit establishment. Soil factors such as aluminum saturation and calcium carbonate content also contributed meaningfully, possibly affecting root development and nutrient availability in acidic or calcareous soils.

### 4.3 Current and future distribution dynamics

Under current climate conditions, the suitable habitat of *B. bipinnata* is largely concentrated in central, eastern, and southwestern China, consistent with its known distribution. The fragmented pattern of high-suitability areas reflects the species’ reliance on specific combinations of climatic and edaphic conditions. Under future scenarios, our models project a general expansion of suitable habitat, particularly toward northern and higher-elevation regions. This shift is more pronounced under the high-emission scenario (SSP5-8.5), where high- and medium-suitability areas expand significantly by the 2070s. In contrast, under SSP1-2.6, the distribution remains relatively stable, with only moderate increases in total area. These patterns are consistent with observations for other temperate medicinal plants, such as *Forsythia suspensa* (Wang et al., 2024) and *Angelica dahurica* (Zhang et al., 2024), which also show northward and upward range shifts under warming climates.

### 4.4 Centroid migration and ecological implications

The centroid of suitable habitat for *B. bipinnata* is currently located in Hubei Province. Under future scenarios, it exhibits directional movement, with more pronounced displacement under SSP5-8.5. This northward and westward trajectory aligns with general responses of plant species to climate warming, as documented in multiple studies across East Asia (Lenoir et al., 2008; Li et al., 2019). However, the assumption of unlimited dispersal in our model likely overestimates the pace of range expansion, particularly in fragmented landscapes or where biotic interactions (e.g., competition, pollination) limit establishment. Future studies should incorporate dispersal constraints and biotic factors to improve realism.

### 4.5 Limitations and future directions

Several limitations should be acknowledged. First, our model relied on a single global climate model (BCC-CSM2-MR); incorporating multiple GCMs could reduce uncertainty. Second, the assumption of unlimited dispersal may not reflect real-world migration barriers. Third, although we reduced spatial autocorrelation, some sampling bias may persist due to uneven herbarium coverage. Finally, factors such as land-use change, biotic interactions, and phenotypic plasticity were not explicitly modeled but could significantly influence the realized distribution of *B. bipinnata*. Future work should integrate these elements to better predict habitat availability under changing environments.

### 4.6 Conservation and cultivation implications

Our findings provide a scientific basis for the conservation and sustainable cultivation of *B. bipinnata*. The projected expansion of suitable areas into northern regions suggests opportunities for introducing cultivation in new areas, such as parts of Shaanxi, Shanxi, and Gansu. However, the loss of suitability in some current marginal areas underscores the need for assisted migration or *ex situ* conservation. Given the species’ ecological and economic value, regional planting plans should consider future climate projections to ensure long-term yield and quality. Moreover, the methodology applied here can be extended to other threatened medicinal plants, supporting the development of climate-resilient cultivation systems for traditional Chinese medicine.

## 5 Conclusion

This study demonstrates that climate change significantly influences the ecological niche and spatial distribution of Bidens bipinnata in China. Using an optimized MaxEnt model, we identified July precipitation and February mean temperature as the pivotal environmental factors constraining its current distribution. Under future climate scenarios, the potential suitable habitat for *B. bipinnata* is projected to expand overall and shift towards northern and higher-altitude regions. This northward and upward shift aligns with documented responses of numerous plant species to global warming.

The findings provide a scientific basis for the ecological cultivation and resource conservation of *B. bipinnata*. The projected expansion of suitability into northern regions suggests potential new areas for cultivation, while highlighting the need to monitor current marginal habitats that may become less suitable. Consequently, region-specific planting strategies should be developed considering these future distribution dynamics, informing the development of climate-resilient cultivation strategies and the conservation of wild medicinal plant resources.

Notwithstanding, this study has limitations, including the use of a single GCM and the assumption of unlimited species dispersal. Future research should incorporate multiple climate models and species-specific dispersal constraints to refine predictions. Field validation in projected expansion zones is also recommended to ground-truth model outputs.

## Data Availability

The original contributions presented in the study are included in the article/**Supplementary Material**. Further inquiries can be directed to the corresponding authors.
