# Supplementary material for: Optimized MaxEnt model predicts potential suitable habitats of Bidens bipinnata in China under climate change scenario
Source: Front Plant Sci. 2025 Dec 16;16:1702523. doi: 10.3389/fpls.2025.1702523 (PMC12750343; doi:10.3389/fpls.2025.1702523)
Supplement: Supplementary file 1 [file Table1.docx]

**Table S1** Detailed information on the 66 environmental variables.

| **Variable code** | **Environmental factor** | **Variable code** | **Environmental factor** |
| --- | --- | --- | --- |
| prec 1-12 | January to December precipitation | alt | Altitude |
| tavg 1-12 | January to December average temperature | slope | Slope |
| zbyl | Vegetation Classification | aspect | Aspect |
| bio1 | Annual Mean Temperature | coarse | Coarse fragments |
| bio2 | Mean Diurnal Range | sand | Sand |
| bio3 | Isothermality | slit | Slit |
| bio4 | Temperature Seasonality | clay | Clay |
| bio5 | Max Temperature of Warmest Month | bulk | Bulk Density |
| bio6 | Min Temperature of Coldest Month | ref_bulk | Reference Bulk Density |
| bio7 | Temperature Annual Range | org_cbn | Organic Carbon Content |
| bio8 | Mean Temperature of Wettest Quarter | ph | pH in water |
| bio9 | Mean Temperature of Driest Quarter | n | Total nitrogen content |
| bio10 | Mean Temperature of Warmest Quarter | cn | Carbon/Nitrogen ratio (C/N) |
| bio11 | Mean Temperature of Coldest Quarter | cec_soil | CEC soil |
| bio12 | Annual Precipitation | cec_clay | CEC clay |
| bio13 | Precipitation of Wettest Month | teb | TEB |
| bio14 | Precipitation of Driest Month | bsat | Base Saturation |
| bio15 | Precipitation Seasonality | alum_sat | Aluminium saturation |
| bio16 | Precipitation of Wettest Quarter | esp | Exchangeable Sodium Percentage |
| bio17 | Precipitation of Driest Quarter | eq | Calcium Carbonate |
| bio18 | Precipitation of Warmest Quarter | gypsum | Gypsum content |
| bio19 | Precipitation of Coldest Quarter | elec_con | Electric Conductivity |
